# In Situ Reconstruction Regenerates Sinter-Degraded NiO-Based Monolithic Ceramic Catalysts for Efficient Methane Oxidation in Ventilation Air

**DOI:** 10.3390/ma19091677

**Published:** 2026-04-22

**Authors:** Fangsheng Liu, Enming Shi, Zhiqiang Cao, Yeqing Wang, Xuemei Ou, Zhen Wang, Xinyi Han, Shiru Le, Zhijiang Wang, Chunlong Cheng, Fangjun Jin

**Affiliations:** 1 School of Materials Science and Physics, China University of Mining and Technology, Xuzhou 221116, China; semhh77@163.com (E.S.); caozq6666@163.com (Z.C.); yeqingwang@cumt.edu.cn (Y.W.); oxm@cumt.edu.cn (X.O.); wangzhen25@cumt.edu.cn (Z.W.); hhhhxxxyyy111@163.com (X.H.); 2 School of Chemistry and Chemical Engineering, Harbin Institute of Technology, Harbin 150001, China; leshiru@hit.edu.cn (S.L.); wangzhijiang@hit.edu.cn (Z.W.); 3 College of Energy Science and Engineering, Huaibei Normal University, Huaibei 235000, China; iolxvxloi@163.com

**Keywords:** NiO/CeO_2_ monolithic ceramic catalyst, in-situ reconstruction, catalytic combustion, high activity, high stability

## Abstract

**Highlights:**

- An in situ reduction–oxidation reconstruction method is proposed to regenerate sinter-degraded NiO-based monolithic ceramic catalysts, effectively reversing NiO agglomeration and coarsening caused by high-temperature sintering.
- The reconstructed catalyst exhibits remarkably boosted activity and long-term durability for methane oxidation in ventilation air, with robust thermal cycling and reversible steam tolerance.
- Structural and interfacial regulation synergistically increases active site density and optimizes mass transfer, offering a feasible route to develop sintering-resistant monolithic ceramic catalysts.

**Abstract:**

Monolithic ceramic catalysts are a key technology for the industrial treatment of coal mine ventilation air methane (VAM). The preparation of straight-channel NiO/CeO_2_ monolithic ceramic catalysts via phase inversion addresses critical bottlenecks for industrial VAM abatement. However, high-temperature sintering leads to irreversible NiO agglomeration and coarsening, severely reducing catalytic activity. In this study, an in situ reduction–oxidation reconstruction method is developed to regenerate sinter-degraded NiO. The reconstructed catalyst increases methane conversion from below 70% after sintering to over 95% at 550 °C and achieves full conversion at 600 °C. The catalyst maintains near 100% conversion during 400 h of continuous operation at 600 °C and shows no performance degradation over 15 thermal cycles. Moreover, the reconstructed catalyst exhibits excellent steam tolerance with fully reversible deactivation. The reconstructed catalyst presents a refined porous structure with BET surface area rising from 4.5 to 11.4 m^2^ g^−1^, an elevated Ni^3+^/Ni^2+^ ratio (1.47 to 1.97), a higher surface adsorbed oxygen proportion (36.8% to 48.7%) and significantly strengthened NiO-CeO_2_ interfacial interaction. This work provides a facile and efficient in situ regeneration strategy, greatly enhancing the VAM oxidation activity and stability of sinter-degraded monolithic ceramic catalysts.

## 1. Introduction

Ventilation air methane (VAM) represents a significant source of greenhouse gas emissions [1,2,3,4,5]. However, its low concentration (~1% CH_4_) makes mitigation particularly challenging [6,7]. Catalytic oxidation significantly lowers the ignition temperature of methane oxidation, enabling high VAM conversion efficiency with reduced energy consumption [8,9,10,11]. This technological advantage positions it as a technically viable and economically attractive solution for VAM treatment. However, the practical application of this technology is critically hindered by the activity and structure stability of the catalysts.

Among various catalyst configurations, traditional powder or pellet catalysts exhibit high initial activity but suffer from poor mass transfer and high pressure drop in fixed-bed reactors [12,13,14,15]. The widely used monolithic honeycomb ceramic reactor offers excellent catalytic activity, low pressure drop, and easy scalability for reactor integration; however, its supported active components are prone to spalling and sintering deactivation under thermal cycling conditions [16,17,18]. Utilizing the excellent C-H bond activation capability of NiO, the development of NiO-based monolithic cermet catalysts provides a new approach to enhancing structural stability [19,20,21]. Monolithic catalysts with micro-nano straight channels, fabricated via mesh-assisted phase inversion, combine efficient mass transfer with high catalytic activity and have garnered considerable attention in the field of electrochemical catalytic fuel oxidation [22,23,24,25,26]. Notably, compositing NiO with CeO_2_, which possesses excellent oxygen storage capacity and redox properties, offers the possibility to construct a highly efficient ceramic catalytic system [14,27,28]. However, high-temperature sintering inevitably causes severe agglomeration and coarsening of the active NiO phase, critically compromising catalytic activity [29,30,31]. Despite the ability to mitigate NiO sintering through precursor optimization, dopant incorporation, or tuning of sintering parameters, such strategies offer only limited relief and fail to disrupt pre-existing dense agglomerates [32,33]. Moreover, existing post-modification methods such as acid washing or secondary loading are often complex and risk damaging the support or introducing new instabilities [34,35].

**Figure 1:**
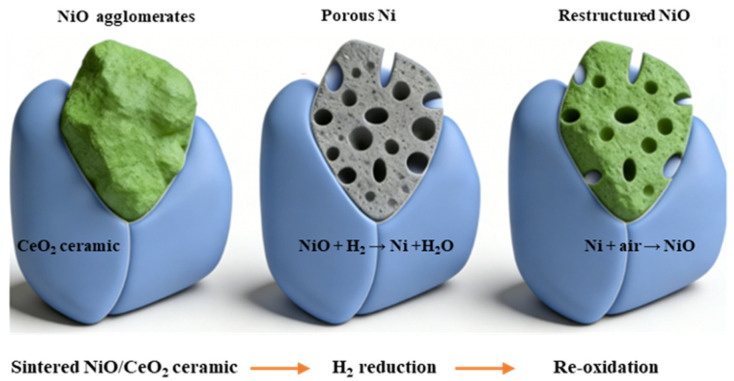
Schematic diagram of the in situ reconstruction process for sinter-degraded NiO/CeO_2_ monolithic ceramic catalysts.

Research indicates that H_2_ reduction can drive the bulk migration of oxygen vacancies via proton permeation, triggering an inside-out metallization and volume contraction of NiO [36,37]. This causes the dense sintered agglomerates to disintegrate and form porous Ni, thereby achieving in situ structural rejuvenation [38,39]. Taking advantage of this feature, the present study proposes an in situ redox reconstruction strategy to regenerate sinter-degraded NiO/CeO_2_ monolithic catalysts. As shown in Figure 1, the dense sintered NiO is first broken down via H_2_ reduction to form a porous metallic Ni intermediate, which is then converted into a regenerated NiO structure with fine particles and open pores through controlled low-temperature oxidation.

The reconstructed monolithic catalysts achieve a significant recovery from sintering-induced performance degradation, with methane conversion rising from below 70% to over 95% at 550 °C and reaching complete conversion above 600 °C. Moreover, the reconstructed ceramic catalyst exhibits excellent structural stability, combining outstanding thermomechanical stability with robust long-term operational durability, maintaining near-complete conversion over 400 h at 600 °C. It also demonstrates superior resistance to steam poisoning and carbon deposition. This work provides a new pathway for developing high-performance, stable VAM catalytic systems.

## 2. Materials and Methods

### 2.1. Materials and Fabrication

NiO/CeO_2_ green bodies were prepared via a mesh-assisted phase-inversion method [40]. A polymer solution was prepared by dissolving polyethersulfone (PES) (Dongguan Jianzhang Plastic Co., Ltd., Dongguan, Guangdong, China) in N-methyl-2-pyrrolidone (NMP) (Aladdin Reagent Co., Ltd., Shanghai, China) at a mass ratio of 17.7 wt%. Subsequently, 60 wt% NiO powder (particle size ~6 μm; Ningbo Suofuren Energy Technology Co., Ltd., Ningbo, Zhejiang, China) and 40 wt% CeO_2_ powder (particle size ~1 μm; Shanghai Macklin Biochemical Co., Ltd., Shanghai, China) were mixed. Then, polyvinylpyrrolidone (PVP, Mw = 40,000) (Sinopharm Chemical Reagent Co., Ltd., Beijing, China) was added at 0.67 wt% relative to the total ceramic powder mass, followed by the addition of the prepared PES solution at 44.3 wt% relative to the total solid content. The mixture was ball-milled (SFM-1, Hefei Kejing Materials Technology Co., Ltd., Hefei, Anhui, China) for 48 h to obtain a homogeneous slurry. The slurry was filtered through a mesh screen, vacuum-degassed (MSK-SFM-7, Hefei Kejing Materials Technology Co., Ltd., Hefei, Anhui, China), poured into a custom mold, and subjected to phase inversion for 2 h to form green bodies. These were immersed in deionized water for 6 h to remove residual NMP, followed by drying at 60 °C. The dried green bodies were then heated in air at 400 °C for 1 h to remove organic components, followed by sintering at 1300 °C for 5 h to obtain sintered monolithic ceramic catalysts. The as-sintered ceramic catalyst was obtained with a diameter of Φ16 × 10 mm. Finally, the button-shaped sintered ceramic catalysts were placed in a tube furnace (KSL-1100X, Hefei Kejing Materials Technology Co., Ltd., Hefei, Anhui, China), heated to 650 °C and purged with N_2_ for 2 h. Then a 15% H_2_/N_2_ gas mixture was introduced for a 5 h reduction. After another N_2_ purge, the catalysts were oxidized with dry air at 650 °C for 3 h, completing the microstructural reconstruction.

### 2.2. Characterization

The microstructures of the ceramic catalysts were characterized by scanning electron microscopy (SEM) (ZEISS, Carl Zeiss AG, Oberkochen, Germany), and energy-dispersive spectroscopy (EDS) mapping was performed using an Oxford Instruments AZtecEnergy system (Oxford Instruments, Abingdon, Oxfordshire, UK). The Brunauer–Emmett–Teller (BET) specific surface area was determined by N_2_ adsorption–desorption at −195.850 °C using an ASAP 2020 Plus HD88 analyzer (Micromeritics Instrument Corp., Norcross, GA, USA), after degassing the sample at 30 °C for 10 min. H_2_-temperature-programmed reduction (H_2_-TPR) was employed to evaluate the reduction behavior. Surface elemental composition and metal chemical states were determined via X-ray photoelectron spectroscopy (XPS) (ESCALAB 250XI, Thermo Fisher Scientific Inc., Waltham, MA, USA, monochromatic Al Kα source). Prior to analysis, the samples were cleaned by Ar^+^ sputtering, and all binding energies were calibrated with reference to the C 1s peak at 284.8 eV. The catalytic performance of the monolithic ceramic catalysts was evaluated in a tubular furnace system equipped with a quartz reaction tube. The button-shaped ceramic catalyst was placed in the isothermal zone of the quartz tube, and fixed at both ends with quartz wool. Simulated VAM with compositions of CH_4_ (2, 3, or 4 vol%), O_2_ (20 vol%), and balance N_2_ was introduced at flow rates of 20, 40, and 60 sccm. The gas flow rate and linear velocity were precisely controlled using a mass flow meter (D07, Sevenstar Electronics Co., Ltd., Beijing, China). The effluent gas components were analyzed online using a gas chromatograph (GC-2014) (Shimadzu Corp., Kyoto, Japan). The CH_4_ conversion rate was calculated based on the concentration difference in CH_4_ between the inlet and outlet streams. The inlet gas was pre-calibrated by gas chromatography to ensure consistency with the designed concentrations.

## 3. Results

### 3.1. Effect of In Situ Reconstruction on the Microstructure

**Figure 2:**
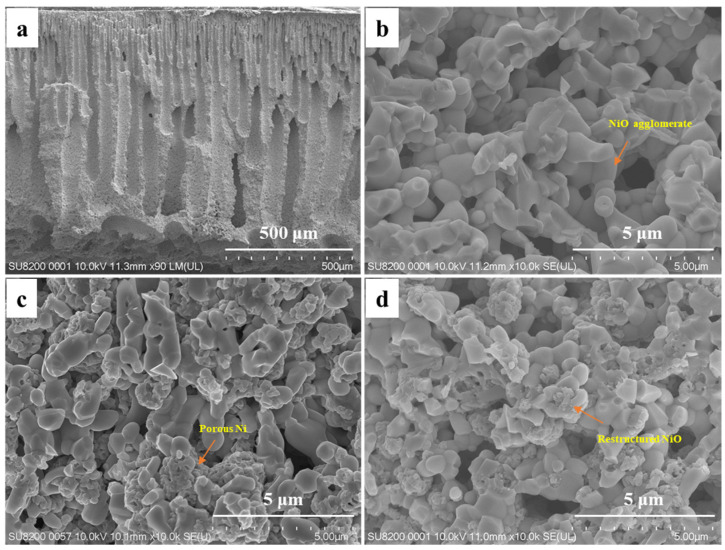
SEM images of NiO/CeO_2_ monolithic ceramic catalysts: (**a**) Cross-section. (**b**) Sintered catalyst. (**c**) H_2_-reduced catalyst. (**d**) Reconstructed catalyst.

As shown in Figure 2a, a NiO/CeO_2_ monolithic ceramic catalyst with straight gradient channels was fabricated by the mesh-assisted phase inversion method, with pore sizes ranging from 1 to 150 μm. The continuous gradient structure delivered excellent mass transfer efficiency and high active interface density, which was advantageous for catalytic reactions requiring efficient gas transport and sufficient active sites [21,23]. However, after high-temperature sintering at 1300 °C, NiO exhibited sintered dense bulk agglomerates. Such agglomeration and coarsening were often irreversible, directly leading to a sharp reduction in the number of active sites (Figure 2b) [41,42]. H_2_ reduction was an in situ regulation method for Ni-based catalysts, which could break NiO agglomerates and construct porous structures in situ to obtain highly dispersed active Ni sites. Accordingly, the densely sintered NiO/CeO_2_ monolithic ceramic catalysts were mildly reduced at 650 °C to realize controllable phase transition and suppress the re-agglomeration and grain growth of active components. As shown in Figure 2c, the reduction process converted dense NiO agglomerates into porous metallic Ni intermediates, completely destroying the original dense structure. The excess oxygen in VAM inevitably led to the oxidation of Ni [43,44]. Directly coupling this oxidation with VAM treatment might release heat, creating local hot spots that caused Ni particles to re-coarsen and agglomerate [45]. To avoid Ni re-coarsening caused by in-reaction hot spots, a mild pre-oxidation at 650 °C was conducted. As shown in Figure 2d, NiO transformed from a coarse sintered morphology into a porous structure with refined particle size after reconstruction. BET results confirmed that after reconstruction, the specific surface area increased from 4.5 to 11.4 m^2^ g^−1^. The in situ reconstruction successfully reversed sintering-induced coarsening and significantly increased the density of accessible active sites.

### 3.2. Effect of Reconstruction on Catalytic Performance

**Figure 3:**
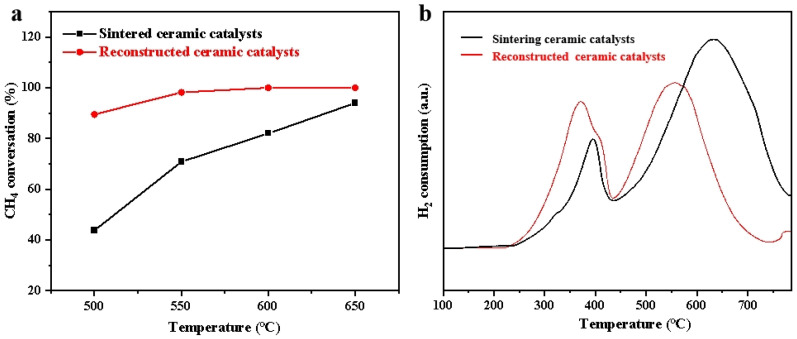
(**a**) Effect of reconstruction on VAM catalytic oxidation performance. (**b**) Reduction properties before and after reconstruction characterized by H_2_-TPR.

Microstructural reconstruction effectively increases the number of active sites, thereby significantly enhancing the intrinsic activity of the catalyst. As shown in Figure 3a, the reconstructed catalyst exhibited greatly enhanced low-temperature catalytic activity toward VAM oxidation. For the feed gas containing 2 vol% CH_4_ at a flow rate of 20 sccm, the CH_4_ conversion increased sharply from 43% to 88% at 500 °C, exceeded 95% at 550 °C, and achieved full conversion above 600 °C. H_2_-TPR results demonstrated that both ceramic catalysts exhibited two reduction features. The low-temperature peak is ascribed to NiO species interacting with CeO_2_, while the high-temperature peak belongs to bulk CeO_2_ (Figure 3b). After reconstruction, both reduction peaks shifted toward lower temperatures, and the intensity of the low-temperature peak increased distinctly, indicating that the reconstruction effectively improved the reducibility of both interfacial NiO species and CeO_2_. This enhancement was mainly attributed to the reconstruction-induced transformation of densely sintered NiO agglomerates into a well-developed porous structure, which greatly enlarged the NiO-CeO_2_ contact interface and the exposed area of active components, thereby accelerating reduction kinetics [14,46].

**Figure 4:**
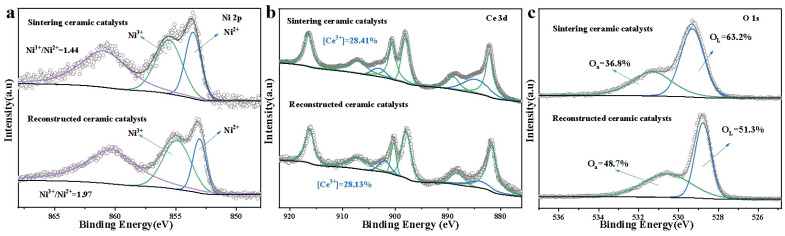
Xps of sintering reactor and reconstructed reactor. (**a**) Ni 2p; (**b**) Ce 3d; (**c**) O 1s.

XPS analysis further revealed the influence of reconstruction on surface chemical states. As shown in Figure 4a, the Ni^3+^/Ni^2+^ ratio rose from 1.47 to 1.97 after reconstruction, indicating more abundant surface defects on NiO with increased high-valence Ni species. These high-valence Ni species were closely related to interfacial defects and promote the initial activation of C-H bonds [47]. The Ce^3+^ proportion remained nearly unchanged, suggesting that the reduced state of CeO_2_ was well maintained (Figure 4b). The proportion of surface-adsorbed oxygen (O_α_) increased prominently from 36.8% to 48.7%, indicating a higher content of reactive oxygen species on the catalyst surface. These species boosted the adsorption and dissociation of gaseous oxygen and participated in the complete oxidation of methane (Figure 4c). Therefore, reconstruction not only improved the exposure of active sites but also induced the enrichment of Ni^3+^ defects and adsorbed oxygen by optimizing the Ni-CeO_2_ interface, thereby synergistically elevating the methane oxidation performance [47,48].

### 3.3. Effect of VAM Input Condition

**Figure 5:**
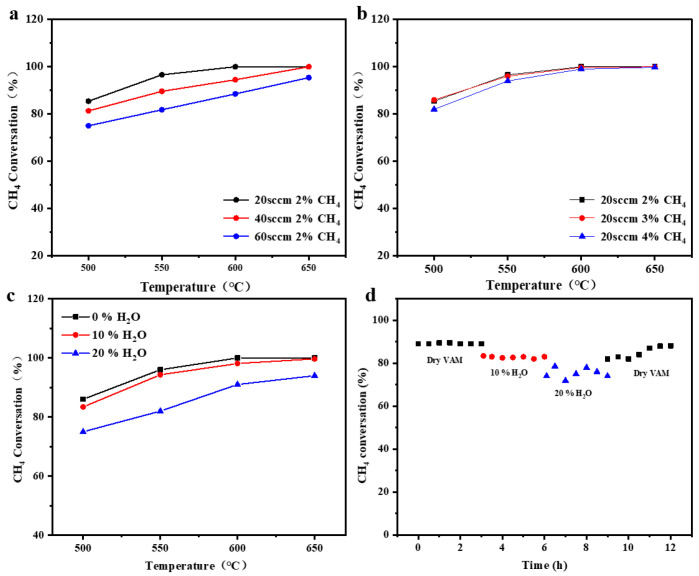
Effect of VAM input conditions on catalytic performance: (**a**) Flow rate. (**b**) Methane concentration. (**c**) Steam content. (**d**) Effect of steam content on stability.

Input condition such as flow rate, methane concentration, and steam content collectively determine the overall efficiency of VAM catalytic oxidation by influencing the reactant diffusion and mass transfer rates as well as the competitive adsorption on the active sites of the catalyst surface. As shown in Figure 5a, methane conversion decreased with increasing flow rate. For example, at 600 °C, the methane conversion rates were 100%, 94.5%, and 88.5% at flow rate of 20, 40, and 60 sccm, respectively. This is because increasing the flow rate limits the diffusion of reactants to the pore walls, preventing them from effectively participating in the reaction and resulting in a decrease in conversion. For practical VAM abatement, the methane concentration must be strictly controlled to remain below the lower explosive limit (LEL) of 5%. Therefore, this study evaluated three representative concentrations (2%, 3%, and 4%). As shown in Figure 5b, increasing methane concentration from 2% to 3% had negligible impact on conversion, while a noticeable decrease occurred only below 550 °C even at 4%. This is due to the unique straight-channel structure and abundant active sites of the reconstructed monolithic ceramic catalyst, which enable efficient catalytic oxidation of methane, thereby rendering methane conversion largely insensitive to moderate increases in methane concentration [10,35,46]. To evaluate the influence of steam, experiments were conducted by introducing 10% and 20% steam into the feed gas composed of 2% CH_4_ at a flow rate of 20 sccm. As shown in Figure 5c, methane conversion decreased slightly at 10% steam concentration, but was significantly suppressed at 20%. For example, at 550 °C, methane conversion dropped from 96% with no steam to 94.3% with 10% steam, and further declined to 82% when the steam concentration was increased to 20%. Stability tests showed minor fluctuations at 10% steam and more pronounced variations on 20% (Figure 5d). The activity fully recovered after ceasing steam introduction, indicating that deactivation was caused by reversible competitive adsorption rather than irreversible structural damage to the catalyst.

### 3.4. Durability

**Figure 6:**
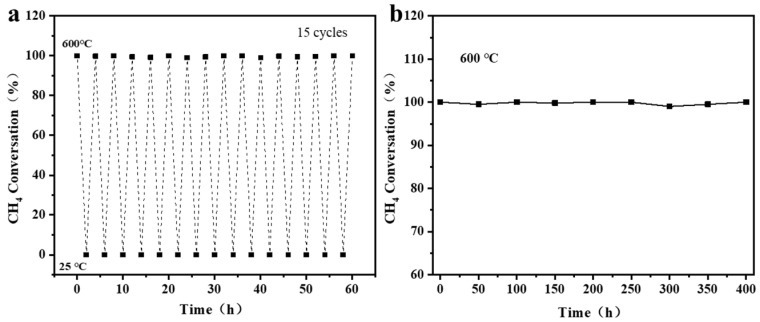
The reconstructed monolithic ceramic catalysts: (**a**) Thermal cycling tests. (**b**) Microstructure of the reconstructed reactor after cycling.

**Figure 7:**
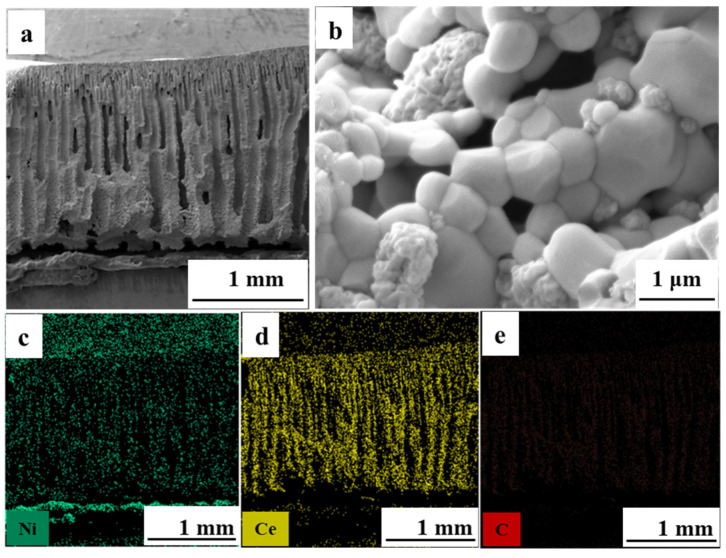
Cross-sectional SEM images (**a**,**b**) and corresponding EDX elemental mappings of the region in panel (**a**) for the ceramic catalyst after 400 h stability test (**c**–**e**).

In practical VAM treatment, monolithic ceramic catalysts must endure frequent start-stops and long-term operation [49]. The minimum temperature for complete oxidation of typical VAM (2% CH_4_, 20 sccm) was determined as 600 °C. Therefore, thermal cycling and long-term stability tests were conducted at this temperature. As shown in Figure 6a,b, during 15 thermal cycles at a ramp rate of 5 °C min^−1^ and over 400 h of continuous operation, the methane conversion for VAM oxidation remained consistently near 100%. SEM images of the tested samples showed that the framework of reconstruction reconstructed monolithic ceramic catalyst was intact (Figure 7a), with no agglomeration or sintering of NiO and intimate contact at the NiO-CeO_2_ interfaces (Figure 7b). Moreover, EDS mapping confirms uniform elemental distribution and the absence of carbon deposition (Figure 7c–e). Through a mild reduction–oxidation process, the sintered NiO particles were effectively refined and reconstructed into a porous structure, while the original ceramic framework remained intact. The reconstructed porous NiO provides abundant active sites and retains a strong bond with CeO_2_. The unique structure confers excellent sintering resistance and structural robustness on the monolithic ceramic catalyst. It effectively suppresses the migration and coarsening of NiO, thereby maintaining stable high catalytic activity toward VAM oxidation during thermal cycling and long-term operation.

## 4. Discussion

In this study, a straight-channel NiO/CeO_2_ monolithic ceramic catalyst was prepared by phase inversion and applied to the catalytic oxidation of ventilation air methane (VAM). To address the severe agglomeration and coarsening of the NiO active phase during high-temperature ceramization, which leads to significant catalytic degradation, an in situ reduction–oxidation reconstruction strategy was developed to regenerate the sinter-degraded active phase.

This strategy is based on the deep reduction mechanism of bulk NiO by H_2_. At 650 °C, the dense NiO agglomerates formed by sintering at 1300 °C are reduced to metallic Ni, accompanied by in situ etching to form a porous skeleton, thereby refining the active phase. Subsequently, controlled oxidation at the same temperature converts the porous Ni into a NiO structure with fine particles and open pores. This process effectively reverses the grain coarsening caused by high-temperature sintering. The BET surface area increased from 4.5 to 11.4 m^2^ g^−1^, and the NiO-CeO_2_ interfacial interaction is significantly strengthened. Meanwhile, the Ni^3+^/Ni^2+^ ratio increased from 1.47 to 1.97, and the surface-adsorbed oxygen proportion rises from 36.8% to 48.7%, indicating enriched surface defects and active sites, which accelerated C-H bond activation and oxygen migration/reduction kinetics.

After reconstruction, the catalyst showed greatly enhanced VAM oxidation performance. At 550 °C, methane conversion increased from below 70% after sintering to over 95%, and complete conversion was achieved at 600 °C. The catalyst maintained nearly 100% conversion after 400 h of continuous operation at 600 °C and after 15 thermal cycles, with no obvious degradation. Steam tests indicated that only reversible competitive adsorption was caused by steam, with no damage to the catalyst structure. The activity was fully recovered after steam removal. Post-test characterization revealed an intact catalyst framework, no obvious agglomeration of the NiO active phase, a strong NiO-CeO_2_ interface, and no detectable carbon deposition.

The integrated design of the straight-channel ceramic skeleton with active catalysts effectively suppresses the detachment and secondary sintering of the active phase. In this study, the in situ reduction–oxidation reconstruction regeneration method proposed offers advantages including simple operation, no damage to the support, broad applicability, and significant performance improvement. This method provides a new route for regenerating sinter-deactivated monolithic catalysts and shows great application prospects.

## Data Availability

The original contributions presented in this study are included in the article. Further inquiries can be directed to the corresponding authors.
